# Supplementary material for: Endocannabinoid dynamics gate spike-timing dependent depression and potentiation
Source: eLife. 2016 Feb 27;5:e13185. doi: 10.7554/eLife.13185 (PMC4811336; doi:10.7554/eLife.13185)
Supplement: Supplementary File 1. — DOI: http://dx.doi.org/10.7554/eLife.13185.017 [file elife-13185-supp1.pdf]

Supplementary file 1A: Parameters of the mathematical model – intracellular dynamics

Description	Name	Values	Units	Reference
I_{NMDAR} to Ca flow conversion	ξ_{NMDAR}	98	µM/pCol	set to match Ca2+ amplitudes in [1]
I_{VSCC} to Ca flow conversion	ξ_{VSCC}	140	µM/pCol	set to match Ca2+ amplitudes in [2]
I_{TRPV1} to Ca flow conversion	ξ_{TRPV1}	290	µM/pCol	set to match Ca2+ amplitudes in [1]
Total endogenous Ca buffer	B_T	4.5	µM	Estimated from our experimental data
Basal cytoplasmic [Ca]	Ca_b	0.1	µM	[1,3]
Time scale for Ca exit	τ_{Ca_b}	0.007	s	Idem
Endogenous Ca buffer affinity	K_{dB}	0.5	µM	[3,4]
ER-to-cytosol volume ratio	ρ_{ER}	0.3	-	Adapted from [4]
IP3R binding rate (inactivation)	a_2	0.5	µM/s	Idem
Maximal SERCA pump rate	v_{ER}	8	µM/s	Idem
IP3R affinity for IP3	d_3	0.9434	µM	[5]
Maximal IP3R rate	r_C	4	1/s	Adapted from [5]
Basal [Ca] in the ER	$Ca_{ER,b}$	65	µM	[6]
Ca leak from the ER	r_l	0.1	1/s	[5]
IP3R affinity for Ca	d_5	0.12	µM	Adapted from [5]
IP3R dissociation constant	d_2	3.049	µM	Idem
SERCA pump affinity for Ca	K_{ER}	0.05	µM	[5]
IP3R affinity for IP3	d_1	0.13	µM	Idem
PLCδ product inhibition	κ_d	1.5	µM	Idem
PLCδ Ca-activation	K_δ	0.1	µM	Idem
5P-IP maximal rate	r_{5P}	0.2	1/s	Adapted from [5]
PI3K maximal rate	v_{3K}	0.001	µM/s	Idem
PI3K Ca-activation constant	K_D	1.5	µM	Idem
PLCδ maximal rate	v_δ	0.02	µM/s	[5]
PI3K affinity for IP3	K_3	1	µM	Idem
Glutamate affinity to mGluR	K_R	1.3	µM	Idem
regulation by PLCβ termination	K_P	10	µM	Idem
PLCβ maximal rate	v_β	0.8	µM/s	Adapted from [5]
PKC Ca-activation constant	K_π	0.6	µM	[5]
Total CaMKIIα concentration	$CaMK_{tot}$	16.6	µM	[7]
Total Calmodulin concentration	CaM_{tot}	0.07085	µM	Adapted from [7]
PKA Hill number	n_{PKA}	3	-	Idem

Referenced articles: [1] Sabatini, B.L., Oertner, T.O. and Svoboda, K. (2002) *Neuron* 33(3):439-452 [2] Carter, A.G. and Sabatini, B.L. (2004) *Neuron* 44(3):483-493 [3] Jackson, M.B. and Redman, S.J. (2003) *J Neurosci* 23:1612–1621 [4] Nägerl, U.V. *et al.* (2000) *Biophys J* 79: 3009–3018 [5] De Pittà M. *et al.* (2009) *J Biol Phys* 35:383-411 [6] Solovyova, N. *et al.* (2002) *EMBO J* 21:622-630 [7] Graupner, M. and Brunel, N. (2007) *PLoS Comput Biol* 3:e221.

Supplementary file 1B: Parameters of the mathematical model – electrophysiology

Description	Name	Values	Units	Reference
TRPV1R max. conductance	g_{TRPV1}	0.0003	nS	Estimated from our experimental data
Permeability of L-type VSCC	p_{VSCC}	0.00000102	µM/s	Value of [1], scaled to ~5,000 spines, radius 1 µm
AMPAR maximal conductance	g_{AMPAR}	5.1	nS	Estimated from our experimental data
AMPAR closing rate constant	β_{AMPAR}	190	1/s	Adapted from [2]
AMPAR opening rate constant	α_{AMPAR}	1.02	1/(µM.s)	Idem
NMDAR maximal conductance	g_{NMDAR}	1.53	nS	Estimated from our experimental data
Magnesium concentration	Mg	1	mM	Directly measured in our experiments
NMDAR opening rate constant	α_{NMDAR}	0.072	1/(µM.s)	Set to emulate the experimental kinetics of NMDAR-transported Ca in [3]
NMDAR closing rate constant	β_{NMDAR}	100	1/s	Idem
Resting membrane potential	V_L	-70	mV	Directly measured in our experiments
Leak conductance	g_L	10	nS	Idem
Membrane capacitance	C_m	0.1	nF	Idem
Extracellular Ca2+ concentration	Ca_{out}	5000	µM	[1]
Duration of depolarization step	DC_{dur}	0.03	s	From the experimental stimulation protocol
Amplitude of depolarization step	$DC_{\max}$	495	pA	Idem
Amplitude of action current	$AP_{\max}$	7020	pA	[4]
Time constant of action current	τ_{bAP}	0.001	s	Idem
Glutamate peak concentration in the cleft	$G_{\max}$	2000	µM	Estimated from our experimental data
Glutamate decay time constant in the cleft	τ_G	0.005	s	Estimated from our experimental data
Delay to bAP outset	δ	0.015	s	From the experimental stimulation protocol
Stimulation frequency	Frequency	1	Hz	From the experimental stimulation protocol

Referenced articles: [1] Wolf, J.A. *et al.* (2005) *J Neurosci* 25(40):9080-9095 [2] Destexhe, A., Mainen, Z., and Sejnowski, T. (1994) *J Comput Neurosci* 1:195-230 [3] Sabatini, B.L., Oertner, T.O. and Svoboda, K. (2002) *Neuron* 33(3):439-452 [4] Fino, E., *et al.* (2010) *J Physiol* 588:3045-3062.

Supplementary file 1C: Parameters of the mathematical model – endocannabinoid dynamics

Description	Name	Values	Units	Reference
Scaling factor for endocannabinoid contribution to plasticity	k_{CB1R}	3000	1/µM	Estimated from our experimental data
AEA efficiency for CB1R activation (compared to 2-AG)	α	0.1	-	Idem
presynaptic plasticity time scale	P_1	1e-9	s	Set to yield rapid / slow changes of Wpre for high / low 2-AG values, respectively
presynaptic plasticity time scale	P_2	1e-5	-	Idem
presynaptic plasticity time scale	P_3	7	-	Idem
presynaptic plasticity time scale	P_4	2	s	Idem
The lower limit of LTD induction	θ_{LTD}^{start}	0.027	-	Estimated from our experimental data
The upper limit of LTD induction	θ_{LTD}^{stop}	0.047	-	Idem
The constant determining the rate of LTD induction	A_{LTD}	0.65	-	Idem
The lower limit of LTP induction	θ_{LTP}^{start}	0.087	-	Idem
The constant determining the rate of LTP induction	A_{LTP}	10.8	-	Idem
CB1R opening rate constant	α_{CB1R}	0.2402	1/(µM.s)	Idem
CB1R closing rate constant	β_{CB1R}	11.072	1/s	Idem
CB1R desensitization rate constant	γ_{CB1R}	416.38	1/s	Idem
CB1R closing rate constant	ε_{CB1R}	0.047796	1/s	Idem
DAGL α affinity for DAG	K_{DAGL}	30	µM	[1]
Maximal rate of MAG lipase	r_{MAGL}	0.5	µM/s	Set for rapid turnover dynamics.
Maximal DAGL α rate	r_{DGL}	20000	µM/s	Idem
Maximal DAG kinase activity	r_{DAGK}	2	µM/s	Idem
Hill number for DAGL activation by Ca	n_c	6	-	Estimated from our experimental data
DAGL dephosphorylation rate	r_P	380	s^{-1}	Idem
DAGL phosphorylation rate	r_K	50	$\mu M^{-1}.s^{-1}$	Idem
Total DAGL concentration	$DAGL$	1	µM	Idem
N-acetyltransferase activity	v_{AT}	0.2	1/s	Idem
FAAH Michaelis-Menten constant	K_{FAAH}	1	µM	[1]
FAAH enzyme activity	r_{FAAH}	4	µM/s	Estimated from our experimental data

Referenced articles: [1] Okamoto, Y. et *al.* (2004) *J Biol Chem* 279:5298-5305.
