# Supplementary material for: Endocannabinoid dynamics gate spike-timing dependent depression and potentiation
Source: eLife. 2016 Feb 27;5:e13185. doi: 10.7554/eLife.13185 (PMC4811336; doi:10.7554/eLife.13185)
Supplement: Supplementary File 2. — DOI: http://dx.doi.org/10.7554/eLife.13185.018 [file elife-13185-supp2.pdf]

Supplementary file 2: Modeling smooth thresholds for eCB-dependent plasticity

The function *Ω* in eq. (1) of the main text gives the direction of plasticity (increase or decrease) depending of eCB levels. This function has the advantage of simplicity but its sharp thresholds might arguably been considered of limited physiological relevance. We thus tested the model output when the threshold of *Ω* in eq. (1) are replaced by smooth ones. We used the following alternative definition for eq.(1)'s *Ω*:

$$\Omega\left(y_{CB1R}\right) = 1 + A_{LTD}\left[\frac{\left(y_{CB1R} - y_0\right)^2}{k_{\varpi}^2\left(y_{CB1R}\right) + \left(y_{CB1R} - y_0\right)^2} - 1\right] + \frac{A_{LTP}}{1 + \exp\left(\dfrac{\Theta_{LTP}^{start} - y_{CB1R}}{0.0013 k_S}\right)} \quad \text{(S1)}$$

where

$$k_{\varpi}\left(y_{CB1R}\right) = 0.004 k_S + 0.01 k_S \left(1 - \frac{\left|y_{CB1R} - y_0\right|}{y_0 - \Theta_{LTD}^{start}}\right)^2 \Pi\left(y_{CB1R}, \Theta_{LTD}^{start}, \Theta_{LTD}^{stop} - \Theta_{LTD}^{start}\right) \quad \text{(S2)}$$

and $y_0 = 1/2\left(\Theta_{LTD}^{stop} + \Theta_{LTD}^{start}\right)$. In this equation, y_0 sets the value of y_{CB1R} for which LTD is maximal, and the function is the step function defined in eq.(15) of the main text. In this version of *Ω*, the sharpness of the transition between the domains (e.g. no plasticity and LTD) is controlled by parameter k_S (smaller values of k_S yield sharper thresholds). The graph of this function is depicted in Figure 4 - figure supplement 1B for $k_S = 2$. With such smooth threshold, the model output is very similar to the sharp threshold case.
